# Delayed diagnosis and treatment of extreme hypertriglyceridemia due to rejection of a lipemic sample

**DOI:** 10.11613/BM.2021.021002

**Published:** 2021-04-15

**Authors:** Jan Van Elslande, Samira Hijjit, Katrien De Vusser, Michel Langlois, Björn Meijers, Ann Mertens, Bart Van der Schueren, Glynis Frans, Pieter Vermeersch

**Affiliations:** 1 Clinical Department of Laboratory Medicine, University Hospitals Leuven, Leuven, Belgium; 2 Clinical department of Nephrology, University Hospitals Leuven, Leuven, Belgium; 3 Department of Laboratory Medicine, AZ Sint-Jan Brugge, Belgium; 4 Clinical Department of Endocrinology, University Hospitals Leuven, Leuven, Belgium; 5 Nutrition & Obesity Unit, Clinical and Experimental Endocrinology, Department of Chronic Diseases, Metabolism and Aging, KU Leuven, Leuven, Belgium.; 6 Department of Cardiovascular Sciences, KU Leuven, Leuven, Belgium

**Keywords:** extra-analytical phase, hypertriglyceridemia, interferences, lipemia, lipoprotein metabolism

## Abstract

**Introduction:**

Most laboratories routinely determine haemolysis, icterus and lipemia indices to identify lipemic samples and reject potentially affected results. Hypertriglyceridemia is the most common cause of lipemia and severe hypertriglyceridemia (≥ 11.3 mmol/L) is a major risk factor of acute pancreatitis.

**Laboratory analysis:**

A 56-year-old woman attended the outpatient clinic for a follow-up visit 1 month after a kidney transplantation. Her immunosuppressive therapy consisted of corticosteroids, cyclosporine, and mycophenolic acid. The routine clinical chemistry sample was rejected due to extreme lipemia. The comment “extreme lipemic sample” was added on the report, but the requesting physician could not be reached. The Cobas 8000 gave a technical error (absorption > 3.3) for the HIL-indices (L-index: 38.6 mmol/L) which persisted after high-speed centrifugation. The patient was given a new appointment 2 days later. The new sample was also grossly lipemic and gave the same technical error (L-index: 35.9 mmol/L).

**What happened:**

The second sample was manually diluted 20-fold after centrifugation to obtain a result for triglycerides within the measuring range (0.10–50.0 mmol/L). Triglycerides were 169.1 mmol/L, corresponding to very severe hypertriglyceridemia. This result was communicated to the nephrologist and the patient immediately recalled to the hospital. She received therapeutic plasma exchange the next day and did not develop acute pancreatitis.

**Main lesson:**

This case illustrates the delicate balance between avoiding the release of unreliable results due to lipemia and the risk of delayed diagnosis when results are rejected. Providing an estimate of the degree of hypertriglyceridemia might be preferable to rejecting the result.

## Introduction

A lipemic blood sample is characterized by increased turbidity, typically caused by hypertriglyceridemia (HTG). The accumulation of the large lipoprotein particles, which are chylomicrons and large very-low density lipoproteins (VLDL), causes absorbance and scattering of light, potentially interfering in spectrophotometric methods and resulting in a “cloudy” sample appearance. Other affected tests include indirect potentiometry (through electrolyte exclusion effect), electrophoresis, and the measurement of hydrophobic analytes such as fat-soluble vitamins and certain drugs (*1*). The overall frequency of lipemic samples ranges from 0.5-2.5%, with outpatient samples typically showing higher frequencies of lipemic samples than samples from hospitalized patients (*2*).

Hypertriglyceridemia is defined as a fasting triglyceride (TG) concentration of > 1.7 mmol/L, with concentrations of 11.3-22.6 mmol/L and ≥ 22.6 mmol/L classified as severe and very severe HTG, respectively (*3*). Hypertriglyceridemia is a common finding in Western society, with one quarter to one third of the adult population having serum TG concentrations > 1.7 mmol/L (*3*). Very severe hypertriglyceridemia, in contrast, is rare with an estimated prevalence of around 0.1% (*4*). A preanalytical cause of severe and very severe HTG is sampling too soon after the intravenous administration of lipid emulsions (*1*). Sampling in a non-fasting state can also increase TG concentrations, but this increase is usually not clinically significant (0.3 mmol/L on average) (*5*). Causes of HTG include diabetes (especially uncontrolled), increased alcohol consumption, metabolic syndrome, certain drugs, nephrotic syndrome, and genetic defects in the triglyceride metabolism (*e.g.,* familial chylomicronemia) (*3*). Triglycerides concentrations > 11.3 mmol/L pose a risk for hypertriglyceridemia-induced acute pancreatitis. Hypertriglyceridemia is the third most common cause of acute pancreatitis, after alcohol and bile stones. The risk further increases when TG concentrations are higher, and values ≥ 22.6 mmol/L warrant immediate treatment to reduce TG (*3*). Very severe HTG is usually caused by a combination of pre-existing primary hyperlipidemia and one or more precipitating factors such as alcohol, the administration of certain drugs (*e.g.*, corticosteroids, immunosuppressants, estrogens), or uncontrolled diabetes (*6*).

Monitoring and management of lipemia in blood samples are handled heterogeneously in laboratories throughout Europe. Most laboratories monitor haemolysis, icterus, and lipemia (HIL) using automated spectrophotometric assays called HIL indices, although visual inspection is also still used (*7*). While the haemolysis index (H-index) closely correlates with haemoglobin content and can be used to determine cell-free haemoglobin (*8*), the lipemia index (L-index) is a measure of turbidity and only weakly correlates with TG concentration (*9*). A recent survey of the European Federation of Laboratory Medicine (EFLM) found that most laboratories reject potentially affected tests when an assay-specific “lipemia-threshold” is exceeded (71.3%), although some laboratories either reject the entire sample (3.8%), release all test results with a specific comment (21.6%) or release all test results without any specific comment (3.3%) (*7*). Since the decision whether or not to release results potentially affected by HIL interference can have a direct impact on patient care, a pragmatic approach is needed. Laboratory medicine professionals should not only focus on the analytical quality of laboratory test results, but also on the clinical consequences of (not reporting) laboratory results (*10*). We present a case report where the clinical diagnosis and treatment were delayed by rejecting of results potentially affected by lipemic interference in a patient with extreme HTG.

## Case description

A 56-year-old woman with a history of Henoch-Schönlein vasculitis and IgA nephropathy developed kidney failure with positive anti-glomerular basal membrane antibodies (Goodpasture syndrome) 3 years ago. Her renal function deteriorated and she required chronic haemodialysis 2 years later. Her medication consisted of methylprednisolone (8 mg/day), gliquidon, estradiol-dydrogesteron (Femoston), bisoprolol, and several painkillers. After a year of dialysis, she received kidney transplantation, which was accompanied by the administration of a single high dose of corticosteroids (540 mg methylprednisolone). Her daily dose of methylprednisolone was temporarily increased to 16 mg per day and treatment with mycophenolic acid, tacrolimus, and trimethoprim/sulfamethoxazole were started.

The patient attended the outpatient clinic for a routine follow-up visit one month after transplantation. She reported symptoms of fatigue and diarrhoea. Venous blood was drawn and sent to the laboratory for routine chemistry, haematology, and coagulation testing. The routine chemistry sample was grossly lipemic and generated a technical error for the HIL-indices (Table 1). Since the error persisted after high-speed centrifugation, the whole sample was rejected with a comment “extremely lipemic sample”. TG measurement was initially not requested on the sample. As prescribed by the laboratory protocol, the laboratory technician tried to reach the treating physician by phone, but the attempt was unsuccessful.

**Table 1:** Selected laboratory results

**Analyte (unit)**	**Day 1** **(1^st^ visit)**	**Day 3** **(2^nd^ visit)**	**Day 3** **(Admission)**	**Day 4**	**Day 4 (pre-plasma-** **pheresis)**	**Day 4** **(post-plasma-** **pheresis)**	**Day 9** **(Discharge)**	**Reference interval**
Triglycerides (mmol/L)	175.9*	168.1*	181.2*	154.18*	123.0*	25.0	19.5	< 1.7
Lipase (U/L)	70*	n.d.	70*	87*	n.d.	147	56	13-60
L-index (mmol/L) After additional centrifugation	38.6 (abs) 33.1 (abs)	35.9 (abs) 32.5 (abs)	Clot error	27.8 (abs) 28.9 (abs)	23.1	4.0	0.6	< 0.1
Sodium (mmol/L)	(113.6)^†^	(110.5)^†^	Clot error	(116.2)^†^	(118.9)^†^	134.5	n.d.	135-145
Sodium BGA (mmol/L)	n.d.	n.d.	n.d.	140	n.d.	n.d.	n.d.	135-145
Creatinine (µmol/L)	(134.4)^†^	(130.8)^†^	Clot error	120.2^‡^	108.7^‡^	84.0	92.8	45.1-84.0
Total cholesterol (mmol/L)	n.d.	n.d.	n.d.	38.6	35.2	n.d.	n.d.	≤ 4.9
Direct LDL cholesterol (mmol/L)	n.d.	n.d.	n.d.	8.7	7.4	n.d.	n.d.	≤ 3.0
**Lipoprotein fractions**								
Alpha (HDL)	n.d.	n.d.	n.d.	n.d.	12.5%	4.9%	n.d.	22-53%
Pre-Beta (VLDL)	n.d.	n.d.	n.d.	n.d.	56.1%	73.3%	n.d.	4-23%
Beta (LDL)	n.d.	n.d.	n.d.	n.d.	8.2%	6.5%	n.d.	38-69%
Chylomicrons	n.d.	n.d.	n.d.	n.d.	23.2%	15.3%	n.d.	0-2%
All results were determined using Roche Cobas c702 (Roche Diagnostics, Basel, Switzerland) except sodium BGA which was determined using ABL 90 FLEX blood gas analyser (Radiometer, Copenhagen, Denmark). *Value obtained after manual 20-fold dilution using 0.9% saline, ^†^value not reported to clinicians, ^‡^accompanied by comment of possible interference by lipemia. BGA – blood gas analysis. L-index – lipemia index. abs - Absorbance > 3.3. n.d. – not determined. HDL – high density lipoprotein. LDL – low density lipoprotein. VLDL – very low density lipoprotein.								

The patient was given a new appointment 2 days later to perform a second blood draw. The new sample generated the same technical errors, and the clinical pathologist was notified by the laboratory technician. A 1:20 sample dilution using 0.9% saline of the original and the new sample was prepared. TG concentration in both samples was > 150 mmol/L (175.9 mmol/L and 168.1 mmol/L respectively). To rule out a possible life-threatening acute pancreatitis, lipase activity was also determined on the 1:20 dilution. Lipase activity was only slightly elevated in both samples (Table 1). The results for TG and lipase were reported, while the other tests were rejected due to lipemic interference. The clinician was notified and the patient was called immediately to the emergency department.

At admission, the patient reported frontal headache in addition to previously mentioned symptoms. She did not have any abdominal pain suggestive of acute pancreatitis and no evidence for acute pancreatitis on imaging. Insulin, glucose, and HCO_3_^-^ infusions were started in the emergency room. Given the extreme HTG, plasmapheresis was performed the next day with therapeutic plasma exchange (albumin 5%) using a Prismaflex system (Baxter, Illinois, USA) with a PF2000N filter. The volume for the exchange was calculated with the Kaplan formula and estimated to be 45 mL/kg (*11*). The plasmapheresis resulted in a drop of TG concentration by 80% from 123.0 mmol/L to 25.0 mmol/L. The patient did not develop acute pancreatitis, although lipase activity rose to a peak of 147 U/L (reference interval: 13-60 U/L) on the day of the plasmapheresis. Since medication was considered the most likely trigger for the extreme HTG, estradiol-dydrogesteron was stopped, trimethoprim/sulfamethoxazole was changed to dapsone, and fluvastatin was started. The dose of methylprednisolone was reduced from 8 mg to 4 mg per day. The patient was discharged after 6 days of hospitalization without any remaining symptoms.

## Laboratory analyses and other diagnostic evaluations

Routine chemistry parameters were determined with a Cobas 8000 c702 analyser (Roche Diagnostics, Basel, Switzerland), complete blood count with Sysmex XE 5000 (Sysmex, Kobe, Japan), and coagulation tests with ACL-TOP 700 (Werfen, Milan, Italy). Point-of-care testing (POCT) of glucose was performed using Accu-Chek (Roche Diagnostics Basel, Switzerland) and blood gas analysis (BGA) with ABL90 FLEX (Radiometer, Copenhagen, Denmark). HIL indices are determined in all routine biochemistry samples on the Cobas c702, using the standard Serum Index Gen. 2 application (10 minutes). After dilution with 0.9% saline, the absorbances at 660 nm (primary wavelength) and 700 nm (secondary wavelength) are used to provide a semi-quantitative estimate of lipemia/turbidity in the sample.

Routine biochemistry samples were collected using BD Vacutainer 4mL lithium heparin plasma tubes with gel separator (PSTII), haematology parameters using BD Vacutainer 4mL K_2_-ethylenediaminetetraacetic acid (EDTA) tubes, and coagulation tests using BD Vacutainer 2.7 mL 0.109 M (3.2%) trisodium citrate tubes (Becton Dickinson, Temse, Belgium). For blood gas collection, electrolyte-balanced heparin (80 IU) coated safePICO aspirators were used (Radiometer, Copenhagen, Denmark). The Roche Cobas 8000 chemistry analyser generated technical error codes Absorbance (abs) > 3.3, Clot, Kin (kinetic), and Proz (prozone) for a majority of requested tests for the first and the second sample for routine biochemistry, including an abs > 3.3 error for the L-index itself. High-speed centrifugation (21,130xg for 10 minutes) was performed, but there was no clear infranatant. The laboratory technician, therefore, rejected all test requests with a comment stating that the sample was “extremely lipemic”. Glucose concentration was 12.5 mmol/L as measured by a POCT device, confirming inadequate glycaemic control. To exclude ionic disturbances, BGA was performed, which showed a slightly elevated potassium concentration (4.6 mmol/L, reference interval 3.5–4.5). Routine haematology showed leukocytosis of 13.6 x10^9^/L (reference interval 4.0-10.0) with neutrophilia, normal platelet count, and increased haemoglobin of 168 g/L (reference interval 120-160 g/L), most likely due to lipemic interference (*12*). The routine haematology results were reported with the addition of a comment (“Potential interference due to extremely lipemic sample”). A urine sample showed mild proteinuria (18 mg/mmol creatinine, reference ≤ 15 mg/mmol), leukocyturia, and haematuria.

TGs were measured using the Roche Triglyceride assay without glycerol blanking. The normal measuring range of the assay is 0.10–10.0 mmol/L with a built-in decrease run using a 1:5 dilution, which increases the measuring range to 50.0 mmol/L. The product insert states that extremely lipemic samples (TG > 33.9 mmol/L)) can produce falsely decreased results, a finding that has been reported in the literature (*13*, *14*). Lipase activity could be measured in the 1:20 dilution due to the measuring range of this assay (3-300 U/L).

Lipoprotein electrophoresis of the blood samples pre-and post-plasmapheresis, as well as the dialysate, was performed by semi-automated agarose gel electrophoresis followed by Sudan Black staining of migrated lipoprotein fractions with the Hydrasys II instrument (Sebia, Vilvoorde, Belgium) using the HYDRAGEL “Lipo + Lp(a)” kit.

## Considered diagnoses and further investigations

First, we ruled out a preanalytical cause, although this was considered less likely since the lipemia was present in two samples collected 48 hours apart. The patient did not receive intravenous lipid emulsions and postprandial lipemia was ruled out since this alone could not explain the very severe HTG in this patient (*5*). The patient reported not to have consumed alcohol.

Very severe HTG is usually caused by a combination of pre-existing primary hyperlipidemia and one or more precipitating factors. Given the patient’s history, uncontrolled diabetes, nephrotic syndrome, and medication were considered as possible precipitating factors. The patient had an elevated blood glucose concentration suggestive of inadequate glycaemic control, but there was no evidence for diabetic keto-acidosis or hyperosmolar hyperglycaemic state. The urine dipstick was negative for ketones and glucose, and BGA (before treatment) revealed a normal pH and bicarbonate. Nephrotic syndrome was excluded since urine total protein was only slightly elevated and plasma albumin concentration was normal. She was taking numerous medications that have been associated with HTG (methylprednisolone, estradiol-dydrogesteron, mycophenolic acid, tacrolimus, trimethoprim/sulfamethoxazole).

A review of the patient’s recent routine laboratory test results revealed she had HTG the day before and the day after her transplantation one month earlier (25.2 mmol/L and 8.8 mmol/L respectively). The patient’s sister was also known with HTG, and her father and two brothers had a history of acute myocardial infarctions between 40 and 50 years of age (with two deaths), suggesting the presence of an underlying genetic predisposition for dyslipidemia. Total and low-density lipoprotein (LDL) cholesterol (measured by a direct LDL assay) were elevated (Table 1) and lipoprotein electrophoresis of the second sample revealed that the predominant lipoprotein particles consisted of a mix of VLDL and chylomicrons, compatible with familial type 5 hyperlipidemia (also called mixed hyperlipidemia). The molar ratio of triglycerides to cholesterol of 3.5:1 on day 4 (pre-plasmapheresis), which is significantly higher than the ratio of 2.2 for TG: VLDL-cholesterol used in the Friedewald formula, also indicates the presence of lipoproteins which are more TG-rich than normal VLDL. Taken together, the acute very severe HTG was most likely caused by a combination of inadequate glycaemic control and drug-induced HTG in a patient with a pre-existing familial hyperlipidemia.

## What happened?

**Figure 1:**
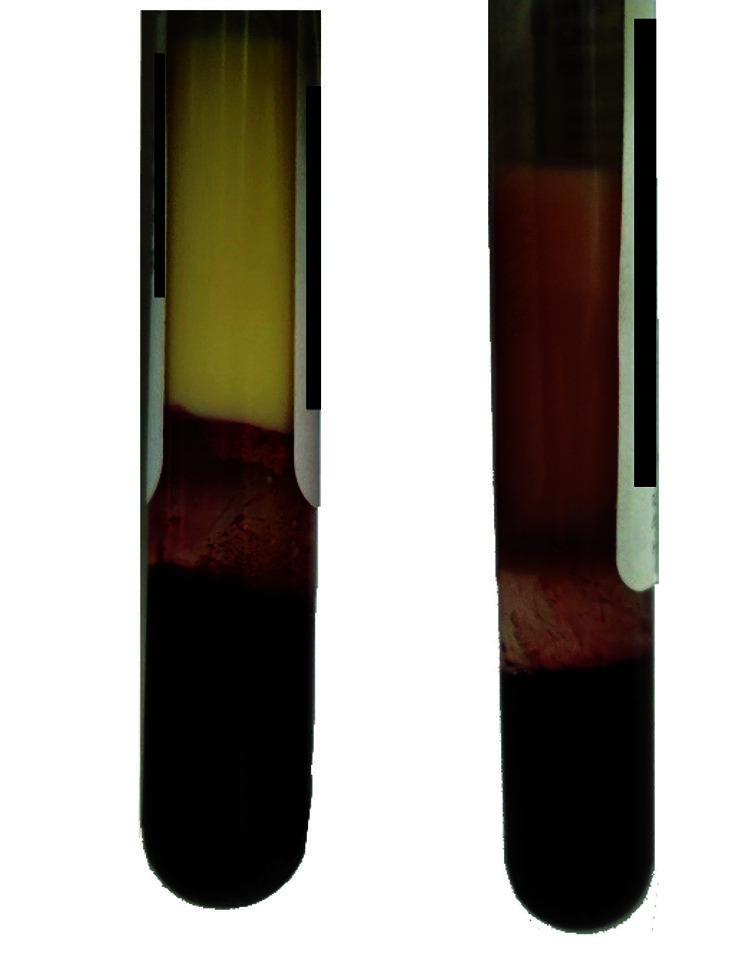
Appearance of blood samples of the patient before plasmapheresis (left) and after plasmapheresis (right). A creamy top layer is visible (arrowhead) in the sample obtained after plasmapheresis.

The extreme lipemia due to very severe HTG generated technical alarm codes for a majority of the requested biochemical tests including the HIL-indices (abs > 3.3), which persisted after high-speed centrifugation. An abs > 3.3 error indicates that the amount of light passing through the reaction cuvette was too low for a reliable photometric measurement because the increased turbidity blocked (almost all) light transmission. The fact that the predominant lipoprotein particles were mostly VLDL rather than chylomicrons (Table 1) explains why the laboratory technician was unable to completely separate the lipoprotein particles from the aqueous phase by high-speed centrifugation (Figure 1). Since the quality of the results could not be guaranteed, the sample for routine biochemistry was rejected with the comment “extreme lipemic sample” as occasionally happens for patients receiving intravenous lipid emulsions. The requesting physician could not be reached by phone as the outpatient clinic was already closed.

Despite the comment on the laboratory test report, the clinician was not immediately alarmed the next morning because in renal transplant patients HTG is common and tacrolimus has been described as an etiological factor (*15*). The patient was given a new appointment for the next day. This led to a delay in treatment of the very severe HTG which could have resulted in acute pancreatitis, a potentially fatal complication. The patient went back home after the second sample was collected and was only recalled and admitted to the emergency department after the clinical pathologist had notified the treating physician about the results of the 1:20 diluted samples for TGs and lipase. In hindsight, the clinician who knew the patient did not receive any intravenous lipid emulsions would most likely have been alerted if he would have had an idea of the magnitude of the HTG. Reporting TG measurements in extremely lipemic samples (TG > 33.9 mmol/L) is, however, not without risk since results can be falsely decreased and grossly underestimated (*13*, *14*).

## Discussion

We present a case report of a patient with extreme HTG after renal transplantation. Tacrolimus is known to cause HTG, although post-transplant hyperlipidemia is typically more pronounced with cyclosporine and sirolimus (*16*). Acute pancreatitis is a feared complication of very severe HTG as it is typically associated with more complications than pancreatitis caused by other aetiologies (*17*). The risk to develop acute pancreatitis rises with increasing TG concentrations, although the severity of pancreatitis does not appear to correlate with TG concentration (*18*). Despite the extreme HTG, our patient did not develop acute pancreatitis, a finding that has also been demonstrated by other case reports (*19*, *20*). The fast initiation of therapeutic plasma exchange may have contributed to preventing acute pancreatitis since a fast reduction of TG concentration is most effective when initiated shortly after presentation (*21*). Apheresis has been described to efficiently reduce TG concentrations by an average of 69% after the first session, in line with the 80% reduction we observed (Table 1) (*22*).

In our laboratory, 0.22% of routine chemistry samples analysed in the past 2 years (2^nd^ October 2018 to 30^th^ September 2020, N = 767,379) had an L-index of > 1.7 mmol/L as measured by the Roche Cobas c702. Most laboratories monitor HIL in blood samples by applying serum indices on automated routine chemistry analysers. The cut-off above which lipemia significantly affects laboratory results, as well as the direction and magnitude of this interference, depends on the analyte, the method used, and also the manufacturer of a specific method (*1*). Manufacturers are urged to provide a cut-off but it is not always clear how this cut-off was established (*23*). Most laboratories rely on the manufacturer method recommendations for acceptable limits and do not verify the manufacturer specified methods (*24*). It is known that these cut-offs are almost always established by using samples spiked with an emulsion (*e.g.*, Intralipid), but these do not necessarily behave in the same way as native lipemic samples (*25*). For example, it has been shown that for intralipid spiked samples, high-speed centrifugation is (almost) equivalent to ultracentrifugation in removing lipids from serum samples (*26*). High-speed centrifugation can separate chylomicrons, however, for patient samples containing mostly VLDL particles, it is not as effective as was illustrated in this case (*1*). The gold standard to analytically manage lipemic samples would be to perform ultracentrifugation, but this technique cannot realistically be implemented in a routine clinical laboratory offering a 24/7 service. Removal of lipids by polar solvents (*e.g.*, Lipoclear) has also been suggested, although studies have shown this technique produces inaccurate values for several assays (*1*). Finally, prolonged high-speed centrifugation (*e.g.*, 3 hours at 13,000xg) can also be considered in cases like this one (*27*).

**Figure 2:**
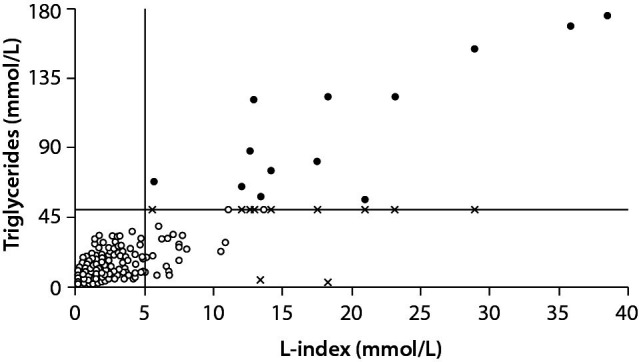
Relationship between L-index and triglyceride concentration (mmol/L) in routine samples in 2018-2020 (N = 88,946). The vertical line corresponds to a L-index of 5.1, while the horizontal line corresponds to the upper limit of the measuring range of the Roche triglyceride assay (50.0 mmol/L) on Cobas c702 analyser (Roche Diagnostics, Basel, Switzerland). Severely haemolytic samples above the Roche-designated threshold for interference were excluded (H-index > 700). The graph includes reported results from Cobas c702 (O), initial results from Cobas c702 (X), later corrected after manual dilution (**·**). L-index – lipemia index. H-index – haemolysis index.

There are different post-analytical strategies across laboratories to deal with potentially biased results by HIL interference. This ranges from rejecting the entire sample with a comment, to releasing all results without indicating possible interference because of lipemia (*7*). We received feedback from the clinicians that a rejected result with a comment stating “extreme lipemia” is not perceived as alarming compared to reporting an estimated value of the actual triglyceridemia. To measure TGs in all severely lipemic samples while avoiding reporting grossly underestimated results, we implemented a new algorithm in our laboratory. If the L-index exceeds 5.1 mmol/L, the sample must be manually diluted 1:20 by the laboratory technician. In the past 2 years, this algorithm would have resulted in the manual dilution of 34 of the 88,976 samples (0.04%) with a request for TGs and an L-index exceeding 5.1 mmol/L (Figure 2). One of these 34 samples, from a patient with very severe HTG, had a grossly underestimated TG result. The Cobas c702 gave a result of 4.6 mmol/L without any technical errors for the TG assay or the HIL-indices despite an L-index of 13.4 mmol/L. A 1:20 manual dilution, however, gave a TG result of 58.3 mmol/L, illustrating that extremely lipemic samples can indeed produce falsely decreased results (*13*, *14*). We now also automatically trigger triglycerides as an add-on test if the L-index exceeds 5.1 mmol/L. This would have resulted in 129 add-on tests of 767,378 samples (0.017%) over the same two-year period. Of note, the European In Vitro Diagnostic (IVD) Regulation 2017/746 will require that modifications to commercial IVD methods should be validated by the laboratory and registered as a lab-developed test (*28*). It remains unclear whether this will also apply to manual dilution of samples for results above the upper limit of the measuring range (*e.g.*, creatine kinase in rhabdomyolysis, ferritin in hemophagocytic lymphohistiocytosis). This case illustrates the delicate balance between avoiding the release of results biased by HIL interference and the risk that rejecting samples because of HIL interference can delay the diagnosis.

## What you can do in your laboratory to prevent such errors

In case of a technical alarm code due to extreme lipemia, measuring TG concentration after manual dilution is preferable to rejecting the sample with a comment “extreme lipemic sample”
Manual dilution of severely lipemic samples (L-index > 5.1 mmol/L)) can help prevent the release of falsely decreased TG results.
